# Predicting the course of asthma from childhood until early adulthood

**DOI:** 10.1097/ACI.0000000000000810

**Published:** 2022-02-03

**Authors:** Hans Jacob L. Koefoed, Judith M. Vonk, Gerard H. Koppelman

**Affiliations:** a Department of Pediatric Pulmonology and Pediatric Allergology, Beatrix Children's Hospital; b Groningen Research Institute for Asthma and COPD (GRIAC); c Department of Epidemiology, University Medical Center Groningen, University of Groningen, Groningen, the Netherlands

**Keywords:** Asthma, Wheezing, Remission, Persistence, Predictors

## Abstract

To communicate recent insights about the natural history of childhood asthma, with a focus on prediction of persistence and remission of childhood asthma, up to early adulthood.

**Recent findings:**

Lung function around the age of 8–9 years is the strongest predictor: obstructive lung function predicts asthma persistence up to early adulthood, whereas normal lung function predicts remission. The ability to predict asthma remission improves when lung function is combined with blood eosinophil levels and degree of bronchial hyperresponsiveness. Interventions, such as inhaled corticosteroids and immunotherapy do not appear to alter the course of asthma. Epigenetic studies have revealed potential novel biomarkers of asthma remission, such as micro-RNA patterns in blood. Specifically, lower serum levels of mi-R221-5p, which is associated with lower IL-6 release and eosinophilic inflammation, predict remission. Higher levels of blood DNA-methylation of a CpG site in *Peroxisomal Biogenesis Factor 11 Beta* were associated with asthma remission.

**Summary:**

Lung function, allergic comorbidity and polysensitization in childhood predict the course of asthma. Recent epigenetic studies have provided a better understanding of underlying pathological processes in asthma remission, which may be used to improve prediction or develop novel treatments aimed at altering the course of asthma.

## INTRODUCTION

The clinical syndrome of childhood asthma is characterized by common symptoms, such as wheezing, shortness of breath, chest tightness and cough in combination with variable expiratory flow limitation and bronchial hyperresponsiveness (BHR) [1,2]. It has become increasingly clear that these symptoms arise from different (inflammatory) subtypes, making asthma a syndrome rather than one disease [3]. Variation exists not only in asthma subtypes but also in the course of asthma, from patients that experience complete remission to those with persistent, sometimes even life-long symptoms.

Although considered a chronic disease in childhood, the course of asthma varies. Almost 80% of patients with asthma experience symptoms during the first 6 years of life [4,5]. In children with asthma at the age of 7 years, 67–75% will be symptom free as adults [6–8]. In contrast, research suggests that approximately 3–5% of the general population will experience persistence of symptoms from childhood until early adulthood, accompanied by low lung growth [6,8–10,11^▪^]. In a population-based birth cohort followed until age 24 years, 6.4% of all children had adolescent-onset asthma with symptom onset after age 8 [11^▪^]. However, remission of symptoms may not reflect disappearance of underlying disease. Additionally, recall bias of childhood asthma could make it more difficult to interpret asthma trajectories [8]. Ethnicity, allergic comorbidity and baseline disease severity are important predictors of the course of asthma [12–14]. Investigation of factors associated with pediatric asthma persistence and remission is important as it not only provides modifiable characteristics that may be used as targets for intervention but also for guidance as to which individuals may or may not require more rigorous clinical follow-up. This review aims to summarize the latest insights into important factors related to the course of asthma from childhood to early adulthood. 

**Box 1:**
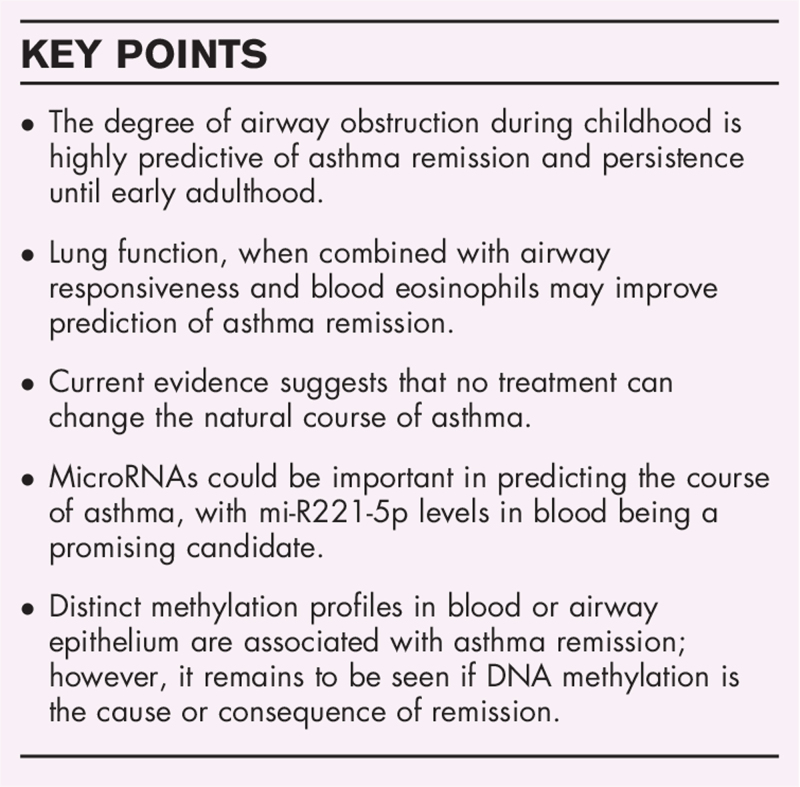
no caption available

## METHODS

We performed a literature search in PubMed to identify studies investigating predictive factors associated with the course of asthma from childhood until early adulthood (Table 1). Backward citation was performed in all selected articles. All included articles were peer-reviewed and published in English. We prioritized articles published in the last 18 months. On the basis of retrieved literature, we selected the following predictors for discussion: lung function, BHR, sex, treatment, allergic comorbidity, genetics, and epigenetics (microRNAs and DNA methylation).

**Table 1:** Search strategy using PubMed

Search strategy We searched PubMed for articles published in the last 2 years using the following key terms: PubMed (MESH terms) Remission, Spontaneous/Remission Induction/Clinical Deterioration/Recurrence/Sex/Age of Onset/Age Distribution/Ethnic Groups/Allergy and Immunology/Hypersensitivity/Immunoglobulin E/Rhinitis, Allergic/Dermatitis, Atopic/Bronchial Hyperreactivity/Genetic Phenomena/Genetics/Biomarkers/Lung Volume Measurements/Respiratory Function Tests/Spirometry/Lung growth and development Title Abstract remission^∗^/persisten^∗^/deterioration^∗^/relapse/recurren^∗^/phenotype^∗^/predict^∗^/clinical outcome^∗^/long-term^∗^/longitudinal^∗^/sex/gender^∗^/age/ethnic^∗^/allerg^∗^/immunol^∗^/hypersensit^∗^/hyperrespon^∗^/Immunoglobulin E/rhinitis/dermatitis/hyperreactiv^∗^/gene^∗^/biomarker^∗^/onset/late/early/intermediat^∗^/transient^∗^/prolonged/endotype^∗^/lung growth/pulmonary growth/lung function meas/spiromet/plethysmograph^∗^/forced oscillation technique^∗^/lung clearance index/multiple breath washout lung function^∗^/pulmonary function^∗^

## THE COURSE OF ASTHMA FROM CHILDHOOD TO EARLY ADULTHOOD

**FIGURE 1:**
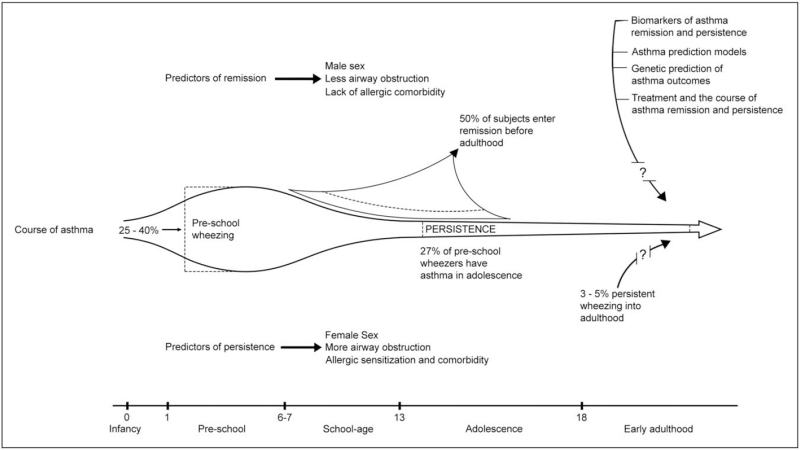
The course of asthma from childhood until early adulthood. Please note that because of recall bias in adulthood, trajectories of asthma persistence and remission may in some cases be misinterpreted.

Approximately 25–40% of all children wheeze during the first 7 years of life and six longitudinal patterns of wheeze in the first years of life have been distinguished with differences in association with asthma later in life (Fig. 1) [15,16]. For example, transient wheeze (wheezing in the first 3 years of life but not thereafter), is not strongly related to asthma later in life. In contrast, persistent (wheezing during the first 6 years of life), intermediate-onset and late-onset wheezing (wheezing starting at 1.5 and 3.5 years, respectively) during childhood has been associated with an asthma diagnosis at school-age. This suggests that these wheezing phenotypes likely reflect onset of conditions collectively identified as asthma [16–18]. Risk factors for these latter phenotypes include aeroallergen sensitization and sex. Before adolescence, male sex is associated with wheezing phenotypes and school-age asthma [19]. However, as puberty progresses, asthma prevalence and severity increase in the female population while the opposite is seen in males [4,20]. Other risk factors for asthma persistence include allergic sensitization during childhood, more severe asthma and a more obstructive lung function between the age of 5 and 12 [21,22]. Allergic sensitization plays an important role in the course of asthma, as suggested by investigations in the EGEA cohort, that reported that poly-sensitization was associated with asthma, allergic comorbidity and lower lung function outcomes [23].

In contrast, key factors associated with remission of asthma until early adulthood are milder initial disease, lack of allergic sensitization and comorbidity, lower degree of BHR and a better lung function [12,24]. Of individuals with persistent asthma during childhood, approximately 30% will have symptomatic (clinical) remission, defined as no recent wheeze or asthma attacks and no use of ICS with an additional 20% going into complete remission, which also includes a normal lung function and absence of BHR [25].

## PREDICTIVE FACTORS FOR THE COURSE OF ASTHMA

### Lung function

A low lung function early in life predicts asthma later in life [26–28]. In addition to being regarded as an outcome of asthma, lung function is also a predictor of asthma remission and persistence [9]. In the Childhood Asthma Management Program (CAMP) study, a 10% higher FEV_1_/FVC during childhood was associated with an odds ratio (OR) of 4.62 for asthma remission during early adulthood (age 18–23 years) [29^▪▪^]. Similarly, fewer than 10% of individuals with a baseline FEV_1_/FVC ratio less than 80% would enter remission [29^▪▪^]. In contrast, of individuals with an FEV_1_/FVC ratio above 90% during childhood, 54% of male individuals and 70% of female individuals would enter remission before early adulthood. These findings were replicated in two Dutch cohorts, showing that FEV_1_/VC measured at school age was significantly higher in individuals who later went into remission compared with individuals with persistence of asthma in adulthood [30]. In the CAMP cohort, of individuals with a FEV_1_/FVC less than 70%, only 1.4 and 11.1% of male and female individuals would enter asthma remission thereby being highly predictive of asthma persistence [29^▪▪^]. Similarly, a 5% lower post-bronchodilation FEV_1_/FVC was associated with an OR of 2.36 for persistence of a severe asthma phenotype until early adulthood [22]. This is contrary to findings from a cohort of individuals with a severe asthma phenotype where neither a low nor an obstructive lung function at school age were predictive of asthma persistence during the 3-year follow-up [31^▪^]. However, longer follow-up is required to assess outcomes in early adulthood.

### Bronchial hyperresponsiveness

BHR, defined as excessive airway constriction resulting from external stimuli is a key hallmark of asthma. In addition to facilitating asthma diagnosis, BHR may predict the long-term course of asthma. Less severe BHR between the ages of 5 and 12 years of age (defined by a higher methacholine PC_20_) was associated with a higher odds of asthma remission in early adulthood [24,29^▪▪^]. Consequently, a lower degree of BHR during childhood may predict a more benign disease trajectory in a population of individuals with mild-to-moderate asthma.

### Sex

Male children more frequently have asthma during early childhood whereas female children have a greater incidence and persistence of symptoms after puberty [4,12]. In a Swedish population-based cohort followed from mid-childhood until the age of 19 years, male sex predicted asthma remission in children with physician-diagnosed asthma [21]. This is in contrast to recent findings from the CAMP study in BHR-positive children with moderate-to-severe asthma where sex was not a predictor of asthma remission [29^▪▪^]. The predictive value of sex in the course of asthma may, therefore, differ depending on the degree of asthma severity. This was also suggested by investigations in a cohort of children with a severe asthma followed over 3 years: remission of severe asthma was equally likely in both boys and girls during adolescence [31^▪^]. The course of asthma is associated with puberty and sex, likely as the result of sex hormones affecting airway homeostasis and development [20]. Therefore, future research on this topic should employ sex stratification.

### Treatment

Anti-inflammatory therapy with inhaled corticosteroids (ICS) is the cornerstone of asthma treatment. Investigation into the association between asthma treatment modalities and longitudinal outcomes is complicated as therapy regimens change regularly and confounding by indication (start of treatment in more severe cases) may complicate interpretation in observational studies. In the CAMP study, which started as a randomized clinical trial (RCT) comparing ICS with placebo on long-term lung function, treatment with ICS for at least 4 years did not affect asthma persistence or remission later in life [22]. These findings suggest that the role of ICS treatment is limited in affecting asthma outcomes. In the population-based OLIN cohort constituted of asthmatic children enrolled at age 7–8 years, treatment with ICS was associated with persistence of asthma [21]. However, these findings are difficult to interpret when studied in a nonrandomized setting as individuals with more severe asthma have a higher prevalence of ICS use [32,33].

Allergen immunotherapy (AIT) has been associated with better, short-term, clinical outcomes in children with asthma and allergy. Despite this, GINA guidelines do not recommend AIT in children with asthma, partly because of studies employing unvalidated symptom and medication scores [2,34]. It is uncertain to which extent this disease-modifying treatment may alter the course of asthma. In a 3-year natural history study on asthmatic children sensitized to HDMs (house dust mites), the association between 3-year and 5-year courses of specific AIT and asthma remission was reported. Remission was defined as 12 months with no asthma symptoms requiring medication and a negative bronchial provocation test. After 3 and 5 years of treatment, 50 and 54.2% of the treatment group had entered remission as opposed to 3.3% in the control group receiving AIT [35]. These findings suggest that AIT could play a role in control of asthma symptoms and possibly alter the natural course of asthma. In a RCT of 812 pediatric individuals with allergic rhinoconjunctivitis without a history or signs of asthma, despite beneficial long-term clinical outcomes, sublingual immunotherapy (SLIT) did not affect the time to onset of asthma when compared with the control group [36]. This suggests that AIT, despite its therapeutic value for allergic rhinoconjunctivitis, does not prevent asthma. More high-quality, double-blinded, long-term follow-up studies are required to establish if AIT alters the natural evolution of asthma onset and persistence [37].

### Allergic comorbidity

Allergic rhinitis and eczema have been associated with a lower likelihood of asthma remission [38,39]. The role of allergy in asthma persistence was supported by findings from the Swedish OLIN population-based cohort of individuals with asthma where allergic sensitization to furred animals was associated with a lower likelihood of remission in early adulthood [21]. Likewise, the Tasmanian Asthma Study reported that early-onset asthma and allergy phenotypes were associated with worse clinical outcomes, such as a lower and more obstructive lung function later in life in addition to a greater predisposition to Chronic Obstructive Pulmonary Disease (COPD) when compared with individuals with ‘minimal or least asthma and allergies’ [40^▪▪^]. Consequently, allergic sensitization and the presence of allergic comorbidity are risk factors for persistence of asthma. Future research should investigate the effect of targeted treatment of allergic comorbidities, including biologicals, on longitudinal asthma outcomes.

### Genetic factors

The pathogenesis of asthma is a complex interplay between environmental and genetic factors. Findings from a genome-wide association study (GWAS) have shown that over 100 genetic loci may be associated with the development of childhood and adult-onset asthma [41]. However, limited research has been performed on genetic prediction of asthma persistence and remission. A GWAS in a Dutch hospital-based cohort constituted of 790 asthmatic patients followed from preadolescence of which 179 individuals were in clinical remission and 55 individuals were in complete remission investigated single nucleotide polymorphisms (SNPs) associated with remission [42]. Replication was performed in two independent cohorts followed by expression quantitative loci analysis (eQTL) in lung tissue for identified SNPs. For complete remission, three SNPs were replicated with one (rs6581895) almost reaching genome-wide significance. This SNP was further identified as an eQTL for fibroblast growth factor receptor 2 (FRS2) and for chaperonin containing TCP1 subunit 2 (CCT2). The association between FRS2 and asthma remission may suggest that this gene is involved in resolution of inflammation [43]. Another SNP (rs1420101) associated with complete remission, was a cis-eQTL for *IL-1RL1* and *IL-18R1*[43]. The childhood asthma susceptibility gene *IL-1RL1,* which encodes the receptor of the epithelial alarmin IL-33, is known to promote airway inflammation [44,45]. More research is needed on variation of *IL-1RL1* in different asthma phenotypes to establish, which individuals may benefit from treatments, such as anti-IL 33 [46]. Recently, a phase II clinical trial with anti-*IL-1RL1* (astegolimab) showed that this drug significantly reduced asthma exacerbations in a broad population of patients, with inadequately controlled, severe asthma [47].

### Epigenetic factors: microRNAs

MicroRNAs (miRNAs), which are small noncoding RNA that have regulatory function, have shown promise as a possible biomarker for predicting the course of pediatric asthma. In a recent study, several blood miRNAs were associated with the risk of asthma exacerbations in the CAMP cohort [48]. In the same cohort, miRNAs could also predict asthma remission [49^▪^]. In the multivariate analysis, a higher blood expression of mi-R221-5p at baseline was associated with a lower likelihood of asthma remission by early adulthood. In addition to promoting IL-6 release and IgE-mediated mast cell degranulation, higher mi-R221-5p expression has been linked to higher eosinophilic airway inflammation [50,51]. Therefore, blood mi-R221-5p levels could serve as a potential biomarker for asthma remission.

The role of miRNA expression in individuals with different asthma outcomes has also been studied in adult populations [52^▪^]. In a recently published Dutch study, total RNA was obtained from bronchial biopsies in 14 individuals with complete asthma remission, 46 patients with persistent asthma and 82 healthy controls. When comparing complete remission to persistent asthma and complete remission to healthy controls, 10 and 77 miRNAs were differentially expressed. Furthermore, by applying a Bayesian network analysis, a network of miRNAs and long noncoding RNAs characteristic of complete remission was described. Of interest, this work showed that asthma remission was clearly different compared with a healthy state.

### Epigenetic factors: DNA methylation

**FIGURE 2:**
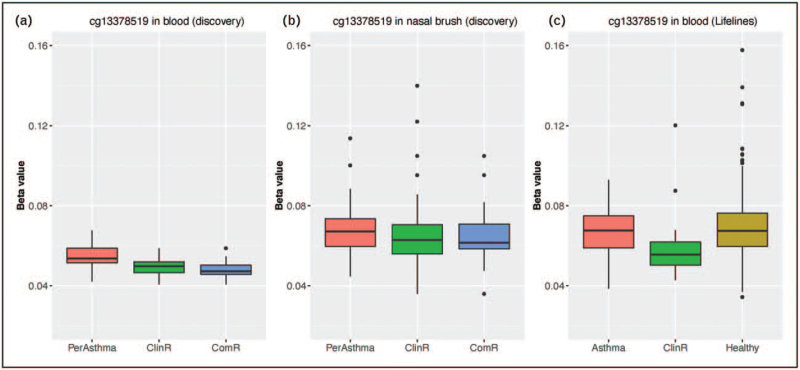
Boxplot illustrating DNA methylation levels of cg13378519 in persistent asthma, clinical remission and complete remission subjects. (a) DNA methylation levels in whole blood in discovery cohort; (b) DNA methylation levels in nasal brushes in discovery cohort; (c) DNA methylation levels in whole blood in replication cohort (Lifelines).

Advancements made within the field of (epi)genomic studies may also provide valuable insights into the pathological mechanisms underlying the course of asthma [53^▪^,54^▪^]. Recent research suggests that DNA methylation, the covalent binding a methyl-group to DNA that may regulate gene transcription, is involved in the persistence and remission of asthma. Four CpG-sites (cytosine-phosphate-guanine) and 42 differentially methylated regions were identified in bronchial biopsies of individuals with asthma remission and persistence [53^▪^]. In another study, Qi *et al.*[54^▪^] performed DNA methylation analyses of nasal brushes and whole blood, of which whole blood DNA methylation was replicated in two separate cohorts. Qi *et al.* identified distinct CpG sites associated with clinical (cg13378519 Chr. 1) and complete asthma remission (cg24788483, Chr. 10). The association of these CpG sites with asthma remission suggests that peroxisome proliferation and Wnt signaling may play a role in asthma remission. In nasal brushes, 25 CpG sites were associated with asthma remission phenotypes; these findings require replication. These findings also emphasize that the methylation profile of individuals having undergone remission differs compared with healthy individuals, thereby suggesting that resolution of symptoms does not result in the return to a methylation profile identical to that of nonaffected individuals. This is illustrated for blood CpG methylation of cg13378519 (Fig. 2). However, it remains to be determined if methylation is the cause or consequence of asthma remission. Future studies should, therefore, adopt a prospective design to evaluate the predictive value of DNA methylation on asthma outcomes.

### Combined prediction models for the course of asthma

**FIGURE 3:**
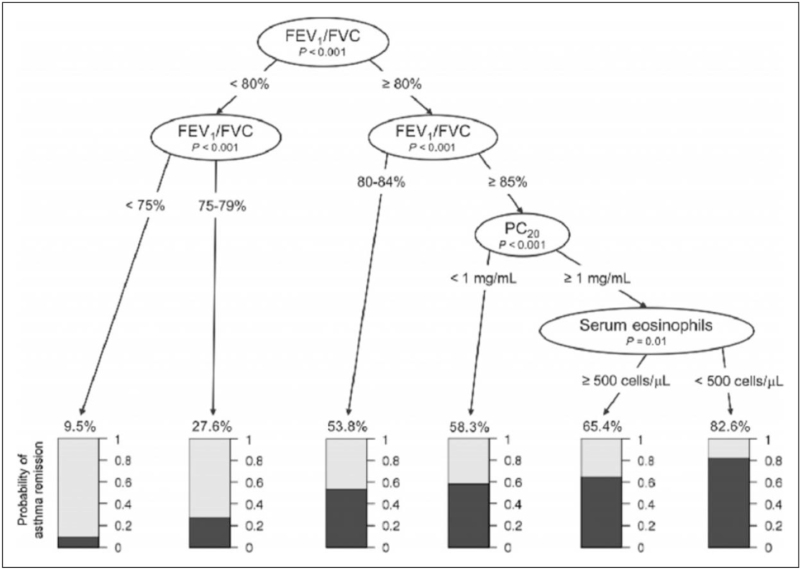
The probability of asthma remission by adulthood based on baseline FEV1/FVC ratio, PC20 and serum eosinophil counts. Reproduced with permission from Wang *et al*. [29^▪▪^]. FEV1/FVC (Forced expiratory volume in 1 second / Forced vital capacity.

Prediction models combine clinical parameters to predict the course of asthma. By combining FEV_1_/FVC (≥ 85%), airway responsiveness (PC_20_ of ≥1 mg/ml) and blood eosinophil measurements (<500 cells/μl) in childhood, Wang *et al.*[29^▪▪^] were able to correctly predict asthma remission in 82.6% of cases until early adulthood (Fig. 3). However, in two Dutch asthma cohorts, this model correctly predicted asthma remission in only 40% of cases [30]. This highlights how combining readily available data may be used to predict future outcomes. To further analyze additional heterogeneity in asthma phenotypes, prediction model development may benefit from incorporating machine learning approaches [55].

## CONCLUSION

An obstructive lung function during childhood, allergic comorbidity and polysensitization are important predictors for persistence of asthma. Improved prediction of persistence is needed to identify patients who require more intense clinical follow-up. Recent epigenetic findings show that asthma remission represents a new state different to that of nonaffected individuals. However, the causality of these findings remains to be determined. The epigenetic studies have provided a better understanding of underlying pathological processes in asthma remission, and this may be used to develop novel treatments aimed at altering the course of asthma.

## Acknowledgements

*None.*

### Financial support and sponsorship

*H.J.L.K., J.M.V. and G.H.K. report grant support from the European Respiratory Society Clinical Research Collaboration (CADSET, Chronic Airway DiSeases Early sTratification).*

### Conflicts of interest

*H.J.L.K. and J.M.V. have no conflicts of interest to report. G.H.K. reports grant support from Netherlands Lung Foundation, TEVA the Netherlands, GSK, Vertex, Ubbo Emmius Foundation, European Union (H2020), outside the submitted work.*

## REFERENCES AND RECOMMENDED READING

Papers of particular interest, published within the annual period of review, have been highlighted as:

▪ of special interest
▪▪ of outstanding interest

## References

[1] Pavord ID Beasley R Agusti A. After asthma: redefining airways diseases. Lancet 2018; 391:350–400. 28911920 10.1016/S0140-6736(17)30879-6

[2] ’Global strategy for asthma management and prevention: 2021 update. 2021. pp. 19–20.

[3] A plea to abandon asthma as a disease concept. Lancet 2006; 368:705. 16935663 10.1016/S0140-6736(06)69257-X

[4] Trivedi M Denton E. Asthma in children and adults-what are the differences and what can they tell us about asthma? Front Pediatr 2019; 7:256. 31294006 10.3389/fped.2019.00256 PMC6603154

[5] Yunginger JW Reed CE O’Connell EJ Melton LJ O’Fallon WM Silverstein MD. A community-based study of the epidemiology of asthma: incidence rates, 1964–1983. Am Rev Respir Dis 1992; 146:888–894. 1416415 10.1164/ajrccm/146.4.888

[6] Strachan DP Butland BK Anderson HR. Incidence and prognosis of asthma and wheezing illness from early childhood to age 33 in a national British cohort. BMJ 1996; 312:1195–1199. 8634562 10.1136/bmj.312.7040.1195 PMC2350975

[7] Jenkins MA Hopper JL Bowes G. Factors in childhood as predictors of asthma in adult life. BMJ 1994; 309:90–93. 8038673 10.1136/bmj.309.6947.90 PMC2540556

[8] Fuchs O Bahmer T Rabe KF von Mutius E. Asthma transition from childhood into adulthood. Lancet Respir Med 2017; 5:224–234. 27666650 10.1016/S2213-2600(16)30187-4

[9] Koefoed HJL Zwitserloot AM Vonk JM Koppelman GH. Asthma, bronchial hyperresponsiveness, allergy and lung function development until early adulthood: a systematic literature review. Pediatr Allergy Immunol 2021; 32:1238–1254. 33835532 10.1111/pai.13516 PMC8453965

[10] Weber P Jarvis D Baptista Menezes AM Goncalves H Duarte de Oliveira P Wehrmeister FC. Wheezing trajectories from childhood to adulthood in a population-based cohort. Allergol Int 2021. 10.1016/j.alit.2021.09.002 34600810

[11▪] Odling M Wang G Andersson N. Characterization of asthma trajectories from infancy to young adulthood. J Allergy Clin Immunol Pract 2021; 9:2368.e3–2376.e3. 33607340 10.1016/j.jaip.2021.02.007

[12] Sears MR. Predicting asthma outcomes. J Allergy Clin Immunol 2015; 136:829–836. 26449797 10.1016/j.jaci.2015.04.048

[13] Melen E Guerra S Hallberg J. Linking COPD epidemiology with pediatric asthma care: Implications for the patient and the physician. Pediatr Allergy Immunol 2019; 30:589–597. 30968967 10.1111/pai.13054

[14] Carpaij OA Nieuwenhuis MAE Koppelman GH. Childhood factors associated with complete and clinical asthma remission at 25 and 49 years. Eur Respir J 2017; 49:1601974. 28596433 10.1183/13993003.01974-2016

[15] Henderson J Granell R Heron J. Associations of wheezing phenotypes in the first 6 years of life with atopy, lung function and airway responsiveness in mid-childhood. Thorax 2008; 63:974–980. 18678704 10.1136/thx.2007.093187 PMC2582336

[16] Savenije OE Granell R Caudri D. Comparison of childhood wheezing phenotypes in 2 birth cohorts: ALSPAC and PIAMA. J Allergy Clin Immunol 2011; 127:1505.e14–1512.e14. 21411131 10.1016/j.jaci.2011.02.002

[17] Lodge CJ Lowe AJ Allen KJ. Childhood wheeze phenotypes show less than expected growth in FEV1 across adolescence. Am J Respir Crit Care Med 2014; 189:1351–1358. 24796409 10.1164/rccm.201308-1487OC

[18] Duijts L Granell R Sterne JA Henderson AJ. Childhood wheezing phenotypes influence asthma, lung function and exhaled nitric oxide fraction in adolescence. Eur Respir J 2016; 47:510–519. 26647439 10.1183/13993003.00718-2015

[19] Granell R Henderson AJ Sterne JA. Associations of wheezing phenotypes with late asthma outcomes in the Avon Longitudinal Study of Parents and Children: a population-based birth cohort. J Allergy Clin Immunol 2016; 138:1060.e11–1070.e11. 27106203 10.1016/j.jaci.2016.01.046 PMC5052126

[20] Fu L Freishtat RJ Gordish-Dressman H. Natural progression of childhood asthma symptoms and strong influence of sex and puberty. Ann Am Thorac Soc 2014; 11:939–944. 24896645 10.1513/AnnalsATS.201402-084OC PMC4213994

[21] Andersson M Hedman L Bjerg A. Remission and persistence of asthma followed from 7 to 19 years of age. Pediatrics 2013; 132:e435–e442. 23897917 10.1542/peds.2013-0741

[22] Izadi N Baraghoshi D Curran-Everett D. Childhood Asthma Management Program Research Group. Factors associated with persistence of severe asthma from late adolescence to early adulthood. Am J Respir Crit Care Med 2021. 10.1164/rccm.202010-3763OC PMC8528529 34029510

[23] Siroux V Boudier A Bousquet J. Trajectories of IgE sensitization to allergen molecules from childhood to adulthood and respiratory health in the EGEA cohort. Allergy 2021. 10.1111/all.14987 34169532

[24] Covar RA Strunk R Zeiger RS. Predictors of remitting, periodic, and persistent childhood asthma. J Allergy Clin Immunol 2010; 125:359.e3–366.e3. 20159245 10.1016/j.jaci.2009.10.037 PMC2844768

[25] Vonk JM Postma DS Boezen HM. Childhood factors associated with asthma remission after 30 year follow up. Thorax 2004; 59:925–929. 15516465 10.1136/thx.2003.016246 PMC1746857

[26] Bisgaard H Jensen SM Bonnelykke K. Interaction between asthma and lung function growth in early life. Am J Respir Crit Care Med 2012; 185:1183–1189. 22461370 10.1164/rccm.201110-1922OC

[27] Malmstrom K Malmberg LP O’Reilly R. Lung function, airway remodeling, and inflammation in infants: outcome at 8 years. Ann Allergy Asthma Immunol 2015; 114:90–96. 25455519 10.1016/j.anai.2014.09.019

[28] Hallas HW Chawes BL Rasmussen MA. Airway obstruction and bronchial reactivity from age 1 month until 13 years in children with asthma: a prospective birth cohort study. PLoS Med 2019; 16:e1002722. 30620743 10.1371/journal.pmed.1002722 PMC6324782

[29▪▪] Wang AL Datta S Weiss ST Tantisira KG. Remission of persistent childhood asthma: Early predictors of adult outcomes. J Allergy Clin Immunol 2019; 143:1752.e6–1759.e6. 30445065 10.1016/j.jaci.2018.09.038 PMC7061344

[30] Carpaij OA Vonk JM Nawijn MC. Applying the CAMP trial asthma remission prediction model to the Dutch asthma remission studies. J Allergy Clin Immunol 2019; 143:1973–1975. 30876728 10.1016/j.jaci.2019.01.040

[31▪] Ross KR Gupta R DeBoer MD. Severe asthma during childhood and adolescence: a longitudinal study. J Allergy Clin Immunol 2020; 145:140.e9–146.e9. 31622688 10.1016/j.jaci.2019.09.030

[32] Chung KF Wenzel SE Brozek JL. International ERS/ATS guidelines on definition, evaluation and treatment of severe asthma. Eur Respir J 2014; 43:343–373. 24337046 10.1183/09031936.00202013

[33] O’Byrne P Fabbri LM Pavord ID. Asthma progression and mortality: the role of inhaled corticosteroids. Eur Respir J 2019; 54:1900491. 31048346 10.1183/13993003.00491-2019 PMC6637285

[34] Normansell R Kew KM Bridgman AL. Sublingual immunotherapy for asthma. Cochrane Database Syst Rev 2015; (8):CD011293. 26315994 10.1002/14651858.CD011293.pub2 PMC6769158

[35] Stelmach I Sobocinska A Majak P. Comparison of the long-term efficacy of 3- and 5-year house dust mite allergen immunotherapy. Ann Allergy Asthma Immunol 2012; 109:274–278. 23010234 10.1016/j.anai.2012.07.015

[36] Valovirta E Petersen TH Piotrowska T. GAP investigators. Results from the 5-year SQ grass sublingual immunotherapy tablet asthma prevention (GAP) trial in children with grass pollen allergy. J Allergy Clin Immunol 2018; 141:529.e13–538.e13. 28689794 10.1016/j.jaci.2017.06.014

[37] Porcaro F Corsello G Pajno GB. SLIT's prevention of the allergic March. Curr Allergy Asthma Rep 2018; 18:31. 29680987 10.1007/s11882-018-0785-7

[38] Burgess JA Matheson MC Gurrin LC. Factors influencing asthma remission: a longitudinal study from childhood to middle age. Thorax 2011; 66:508–513. 21450787 10.1136/thx.2010.146845

[39] To M Tsuzuki R Katsube O. Persistent asthma from childhood to adulthood presents a distinct phenotype of adult asthma. J Allergy Clin Immunol Pract 2020; 8:1921.e2–1927.e2. 31981729 10.1016/j.jaip.2020.01.011

[40▪▪] Bui DS Lodge CJ Perret JL. Trajectories of asthma and allergies from 7 years to 53 years and associations with lung function and extrapulmonary comorbidity profiles: a prospective cohort study. Lancet Respir Med 2021; 9:387–396. 33217367 10.1016/S2213-2600(20)30413-6

[41] Portelli MA Hodge E Sayers I. Genetic risk factors for the development of allergic disease identified by genome-wide association. Clin Exp Allergy 2015; 45:21–31. 24766371 10.1111/cea.12327 PMC4298800

[42] Vonk JM Nieuwenhuis MAE Dijk FN. Novel genes and insights in complete asthma remission: a genome-wide association study on clinical and complete asthma remission. Clin Exp Allergy 2018; 48:1286–1296. 29786918 10.1111/cea.13181

[43] Chen PY Qin L Zhuang ZW. The docking protein FRS2alpha is a critical regulator of VEGF receptors signaling. Proc Natl Acad Sci U S A 2014; 111:5514–5519. 24706887 10.1073/pnas.1404545111 PMC3992672

[44] Saikumar Jayalatha AK Hesse L Ketelaar ME. The central role of IL-33/IL-1RL1 pathway in asthma: from pathogenesis to intervention. Pharmacol Ther 2021; 225:107847. 33819560 10.1016/j.pharmthera.2021.107847

[45] El-Husseini ZW Gosens R Dekker F Koppelman GH. The genetics of asthma and the promise of genomics-guided drug target discovery. Lancet Respir Med 2020; 8:1045–1056. 32910899 10.1016/S2213-2600(20)30363-5

[46] Dijk FN Xu C Melen E. Genetic regulation of IL1RL1 methylation and IL1RL1-a protein levels in asthma. Eur Respir J 2018; 51:1701377. 29519908 10.1183/13993003.01377-2017

[47] Kelsen SG Agache IO Soong W. Astegolimab (anti-ST2) efficacy and safety in adults with severe asthma: A randomized clinical trial. J Allergy Clin Immunol 2021; 148:790–798. 33872652 10.1016/j.jaci.2021.03.044

[48] Li J Panganiban R Kho AT. Circulating microRNAs and treatment response in childhood asthma. Am J Respir Crit Care Med 2020; 202:65–72. 32272022 10.1164/rccm.201907-1454OC PMC7328325

[49▪] Wang AL Li J Kho AT. Enhancing the prediction of childhood asthma remission: Integrating clinical factors with microRNAs. J Allergy Clin Immunol 2021; 147:1093.e1–1095.e1. 32888944 10.1016/j.jaci.2020.08.019 PMC8515417

[50] Perry MM Baker JE Gibeon DS. Airway smooth muscle hyperproliferation is regulated by microRNA-221 in severe asthma. Am J Respir Cell Mol Biol 2014; 50:7–17. 23944957 10.1165/rcmb.2013-0067OC PMC3930931

[51] Xu H Gu LN Yang QY. MiR-221 promotes IgE-mediated activation of mast cells degranulation by PI3K/Akt/PLCgamma/Ca(2+) pathway. J Bioenerg Biomembr 2016; 48:293–299. 27113449 10.1007/s10863-016-9659-7

[52▪] Boudewijn IM Roffel MP Vermeulen CJ. A novel role for bronchial microRNAs and long noncoding RNAs in asthma remission. Am J Respir Crit Care Med 2020; 202:614–618. 32339464 10.1164/rccm.201908-1610LE

[53▪] Vermeulen CJ Xu CJ Vonk JM. Differential DNA methylation in bronchial biopsies between persistent asthma and asthma in remission. Eur Respir J 2020; 55: 10.1183/13993003.01280-2019 31699840

[54▪] Qi C Vonk JM van der Plaat DA. Epigenome-wide association study identifies DNA methylation markers for asthma remission in whole blood and nasal epithelium. Clin Transl Allergy 2020; 10:60. 33303027 10.1186/s13601-020-00365-4 PMC7731549

[55] Kothalawala DM Kadalayil L Weiss VBN. Prediction models for childhood asthma: a systematic review. Pediatr Allergy Immunol 2020; 31:616–627. 32181536 10.1111/pai.13247

